# Unmet Medical Needs Among Immigrants in Korea Before and During COVID-19

**DOI:** 10.3390/healthcare14091226

**Published:** 2026-05-02

**Authors:** Min Young Park, Joonho Ahn

**Affiliations:** 1 Department of Occupational and Environmental Medicine, Seoul St. Mary’s Hospital, College of Medicine, The Catholic University of Korea, Seoul 06591, Republic of Korea; pmy0906@gmail.com; 2 Department of Occupational and Environmental Medicine, Kangbuk Samsung Hospital, Sungkyunkwan University School of Medicine, Seoul 04514, Republic of Korea

**Keywords:** health service accessibility, emigrants and immigrants, healthcare disparities, COVID-19, social determinants of health

## Abstract

**Highlights:**

**What are the main findings?**

- Non-working immigrants in South Korea consistently experienced higher standardized prevalence ratios (SPRs) for unmet medical needs compared to native-born Koreans, a disparity that persisted into the early COVID-19 pandemic.
- While working immigrants showed lower relative risks (SPRs < 1.0), systemic vulnerabilities were most pronounced among male non-workers, those with limited Korean language proficiency, and recent immigrants with shorter durations of stay.

**What are the implications of the main findings?**

- The findings suggest that South Korea’s labor-oriented immigration and health insurance systems create structural barriers for economically inactive immigrants, particularly during public health crises.
- Future healthcare policies must move beyond employment-based coverage to address language access and financial inclusion to ensure equitable health service delivery for marginalized immigrant populations.

**Abstract:**

**Background/Objectives:** This study aimed to investigate how the disparities in unmet medical needs between immigrants to South Korea and native-born populations evolved during the COVID-19 pandemic. **Methods:** Using nationally representative cross-sectional data from the 2018 and 2020 Surveys on Immigrants’ Living Conditions and Labor Force in South Korea, we compared unmet medical needs among immigrants at two time points (N = 12,227 in 2018; N = 18,530 in 2020). Standardized prevalence ratios (SPRs) were calculated. Analyses were stratified according to work status, gender, Korean language proficiency, education level, and duration of stay. **Results:** Working immigrants had lower SPRs for unmet medical needs than Korean nationals (2018: 0.879; 2020: 0.745) but non-workers had consistently higher SPRs (2018: 1.117; 2020: 1.128). The SPRs for male and female non-workers increased and decreased, respectively. The SPRs were persistently higher among individuals with poorer Korean language proficiency, lower education, and shorter duration of stay. **Conclusions:** Systemic disruptions, such as the COVID-19 pandemic, may exacerbate pre-existing healthcare inequalities among immigrant populations. The persistence and widening of these disparities call for targeted policies that address structural barriers and ensure equitable healthcare access during future public health crises.

## 1. Introduction

The health of immigrant populations has emerged as a central public health concern, particularly as in increasing migration rates drive demographic transitions across high-income countries. Immigrants often face disproportionate barriers to healthcare access, including language difficulties, cultural dissonance, lack of insurance coverage, and exclusion from institutional healthcare systems [1,2,3]. Such structural barriers contribute to the consistently elevated rates of unmet medical needs among immigrant populations, as documented in Canada, the United States, and several EU member states [4,5,6,7].

Understanding the disparities requires a theoretical framework that accounts for the interplay between individual characteristics and structural determinants of healthcare access. The Andersen Behavioral Model provides the framework, positing that healthcare utilization is shaped by three domains, predisposing characteristics (sociodemographic traits and health beliefs), enabling resources (insurance coverage, income, and social support), and perceived need [8]. Applied to the immigrant context, this model is particularly instructive to structural conditions such as precarious employment, language limitations, and exclusion from insurance systems systematically constrain enabling resources, thereby amplifying unmet medical needs beyond what individual predisposing factors alone would predict [1,3,8].

South Korea operates a mandatory National Health Insurance (NHI) system that theoretically provides universal coverage; however, enrollment eligibility and contribution structures are closely tied to employment status and visa category [9]. Migrant workers formally employed under the Employment Permit System are enrolled in employer-linked NHI and receive occupational health screenings, whereas economically inactive immigrants or those outside formal employment arrangements face significant institutional barriers to coverage [10,11]. Therefore, non-working immigrants, who are more likely to lack employer-sponsored insurance and to occupy more precarious legal positions, are exposed to compounded structural vulnerabilities in accessing care. Work status, therefore, operates as a central determinant of healthcare access through multiple pathways, such as insurance eligibility, income stability, occupational health screening entitlement, and degree of institutional integration [9,10,11,12,13,14].

Within this framework, work status functions as a primary enabling resource in the Korean context, as it directly determines insurance eligibility under the employment-linked NHI system. Korean language proficiency and education level operate as predisposing characteristics that shape individuals’ capacity to navigate the healthcare system, while duration of stay serves as a proxy for acculturative integration, reflecting progressive shifts in both predisposing and enabling domains over time. Accordingly, the Andersen model predicts that immigrants in Korea, whose enabling resources are systematically constrained by employment-based insurance exclusion, will exhibit substantially higher rates of unmet medical needs than native-born Koreans, and that this disparity will be most pronounced among non-working immigrants with limited language proficiency and shorter duration of stay.

The COVID-19 pandemic has significantly disrupted healthcare systems worldwide and exacerbated access barriers, even in the general population. Emerging evidence from multiple countries documents a pandemic-associated rise in unmet medical needs, with disproportionate increases observed among structurally vulnerable groups [15,16]. For example, a prospective Canadian cohort study reported a marked widening of healthcare access gaps during the pandemic, particularly among those with lower socioeconomic resources [17]. Immigrant populations faced challenges, such as restricted access to testing, vaccination, and primary care, due to language barriers, precarious employment, and low digital health literacy [18]. However, rigorous evidence comparing the magnitude of pandemic-related changes in unmet medical needs between immigrants and native-born populations remains limited, and findings are not entirely consistent across different healthcare contexts.

Unmet medical needs are a widely established indicator of healthcare access and a proxy for health inequalities at the population level [19]. Defined as situations in which individuals are unable to access the required healthcare services despite experiencing symptoms or illness, unmet medical needs have been associated with delayed diagnosis, worsening chronic conditions, and preventable morbidity [8,20,21]. Unlike objective measures of medical service use, unmet medical needs capture perceived barriers to care and offer insights into how individuals experience the healthcare system.

Despite growing global attention to immigrant health, research in Korea remains extremely limited. Recent empirical studies have begun to document structural disadvantages in health and healthcare access among migrant workers in Korea [13,14], but existing studies have primarily relied on objective utilization indicators or focused on specific subgroups [15,22], and none has used nationally representative data to examine how unmet medical needs among immigrants to South Korea evolved across the transition from the pre-pandemic to the early-pandemic period, nor, to our knowledge, has such a temporal comparison been conducted elsewhere in East Asia. This gap matters, as without a pre-pandemic baseline, it is impossible to distinguish pandemic-induced deterioration from the continuation of pre-existing structural inequalities, a distinction with direct implications for whether policy responses should target acute pandemic disruptions or address deeper, structurally embedded barriers that persist independently of external shocks.

Therefore, this study used nationally representative survey data from Korea to examine the disparities in unmet medical needs between immigrants to South Korea and native-born populations. The primary exposure of interest is immigrant status (immigrants compared to native-born Koreans), examined across two time points to assess whether relative vulnerability changed from the pre-pandemic (2018) and early-pandemic (2020) periods. Specifically, this study aimed to (1) evaluate the relative disparities in unmet medical needs between immigrants to South Korea and native-born Koreans across two periods by calculating Standardized Prevalence Ratios (SPRs); (2) assess how disparities differed by work status; and (3) examine subgroup heterogeneity by gender, Korean language proficiency, education level, and duration of stay to identify the most structurally vulnerable immigrant subpopulations. Beyond the COVID-19 context, examining how healthcare access disparities evolve under public health crises may provide important insights into structural resilience and preparedness for future societal shocks.

## 2. Materials and Methods

### 2.1. Participants

**Figure 1:**
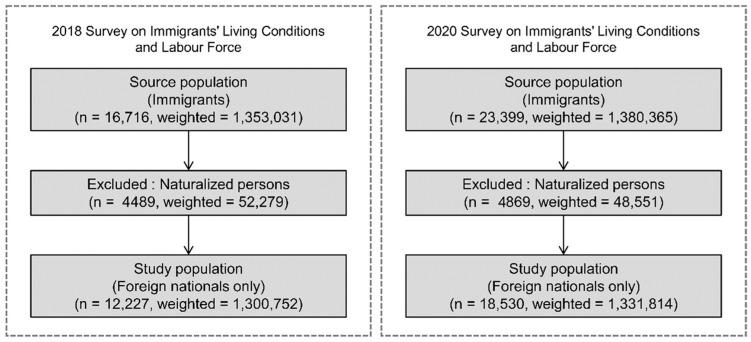
Study population flowchart based on the 2018 and 2020 Surveys on Immigrants’ Living Conditions and Labour Force. Note: ‘n’ represents the unweighted number of respondents, while the weighted count reflects survey weights applied to produce nationally representative estimates.

This repeated cross-sectional study used data from the 2018 and 2020 waves of the Survey on Foreign Nationals’ Living Conditions and Labor Force. We specifically selected these two time points to effectively compare the baseline healthcare access status immediately before the COVID-19 outbreak with the acute impact during the early phase of the pandemic [22,23]. These surveys were conducted jointly by Statistics Korea and the Ministry of Justice and designated as national statistics under the Statistics Act [24]. Stratified random sampling was used across 17 provinces and 90–100 administrative districts. The target population comprised foreign nationals aged ≥15 years residing in Korea for ≥91 consecutive days, which is the legal criterion for registered foreign nationals under the Immigration Control Act, or naturalized citizens within the past 5 years. For this analysis, only foreign nationals were included to focus on individuals retaining foreign nationality under the Immigration Control Act. While the standard epidemiological definition of immigrants includes naturalized citizens, this study operationally defines immigrants strictly as non-citizen foreign nationals. Naturalized persons were excluded to examine the unique structural and legal barriers associated with non-citizenship status, as they possess legal healthcare rights identical to those of native-born Koreans. Notably, there were no missing values for the primary variables (age, gender, and unmet medical needs). The final sample included 12,227 individuals in 2018 (weighted = 1,300,752) and 18,530 in 2020 (weighted = 1,331,814) (Figure 1).

### 2.2. Data Collection

To calculate the SPRs, we used the Korea National Health and Nutrition Examination Survey (KNHANES) as the reference population. KNHANES is a nationally representative survey conducted by the Korea Disease Control and Prevention Agency, employing multistage probability sampling to assess health indicators across the general Korean population [25]. To align survey years, the 2018 immigrant survey was matched with 2018 KNHANES data, and the 2020 immigrant survey with 2020 KNHANES data.

### 2.3. Measures

The outcome was self-reported unmet medical needs, assessed via the question: “Were you ever unable to visit a hospital even though you were sick and needed medical attention in the past one year?”. Responses were dichotomized as having or not having unmet medical needs. This single-item self-reported measure is a universally established and pragmatic indicator widely utilized in major international population-based surveys, such as the European Union Statistics on Income and Living Conditions (EU-SILC), to effectively capture subjective barriers to healthcare access [26,27].

Other variables included work status, Korean language proficiency, education level, and duration of stay. Work status was categorized as workers and non-workers. Korean language proficiency was assessed based on self-rated speaking ability using a 5 point scale. Responses of very well, well, and fair were categorized as good, while poor and very poor were categorized as poor. Education level was dichotomized as high school or less and college or more. Duration of stay was categorized as less than 3 years, 3 to 5 years, and 5 years or more.

### 2.4. Analysis

We used two nationally representative datasets: immigrant surveys (2018, 2020) and KNHANES. To address our primary research question regarding whether immigrants are more vulnerable to unmet medical needs compared to the general population and how this disparity evolved across the pandemic, we calculated SPRs. Analyzing solely within the immigrant survey cannot answer this comparative question. Furthermore, although multivariate regression approaches are widely used, directly pooling the immigrant survey and KNHANES into a single regression model is methodologically inappropriate. The two datasets utilize fundamentally different sampling frames, survey designs, and complex weighting schemes; thus, integration would violate the assumption of variance homogeneity and introduce severe selection bias and distorted standard errors. Therefore, we employed SPR, which is an essential and well-established epidemiological method for evaluating the relative health risks of a specific, hard-to-reach vulnerable population compared to a general reference population when direct pooling is unfeasible [28,29].

The SPR was calculated as the ratio of the observed number of cases in the immigrant population to the expected number of cases. The formal formula is as follows:

$$\mathrm{SPR}\;=\frac{\sum \mathrm{Observed}\;\mathrm{cases}}{\sum \mathrm{Expected}\;\mathrm{cases}}$$

The expected number of cases was derived by applying the age- and gender-specific prevalence rates of unmet medical needs from the reference population (KNHANES) to the corresponding age and gender strata of the immigrant study population. In this process, all prevalence estimates were standardized by gender and age groups of 15–29, 30–39, 40–49, 50–59, and 60 or older. A 95% confidence interval (CI) for each SPR was estimated using the Byar approximation method.

Main analyses were first stratified by work status (workers vs. non-workers) to reflect its policy relevance and potential link to healthcare access [12]. Subsequent subgroup analyses examined differences by gender, language proficiency, education, and duration of stay. SPRs of working and non-working immigrants were compared with working and non-working Koreans, respectively. We aligned groups by age, gender, and education level.

Self-reported reasons for unmet needs were also analyzed by subgroup to aid interpretation. Sensitivity analyses were performed using Poisson-based methods to assess robustness.

Prevalence estimates from the KNHANES were calculated using SAS version 9.4 (SAS Institute Inc., Cary, NC, USA) for accurate handling of complex sample designs, explicitly incorporating strata, primary sampling units (PSUs), and sample weights [30,31]. SAS enables proper treatment of item non-responses without deleting cases, using options, such as NOMCAR [31]. All other analyses, including SPR calculations and visualizations, were performed using the survey package in R version 4.4.3 (64-bit). For the immigrant survey, we applied the survey weights to ensure the estimates accurately represent the target population of registered foreign nationals aged 15 years or older residing in Korea.

### 2.5. Ethical Considerations

The study was conducted in accordance with the Declaration of Helsinki. This study was approved with an exemption of ethical deliberation by the Institutional Review Board of Kangbuk Samsung Hospital, Seoul, Korea (File No. KBSMC IRB 2025-06-003).

## 3. Results

Supplementary Table S1 presents the characteristics of immigrants in Korea according to their experiences of unmet me dical needs in 2018 and 2020. In both years, non-workers reported a higher prevalence of unmet needs (2018: 9.8%; 2020: 7.5%) than workers (2018: 6.9%; 2020: 5.8%). The prevalence of unmet needs was higher among women (2018: 9.1%; 2020: 7.2%) than among men (2018: 6.9%; 2020: 5.8%). The prevalence was also higher among those with poor Korean language proficiency (2018: 9.5%; 2020: 8.1%) than among those with good proficiency (2018: 7.4%; 2020: 5.9%). The prevalence of unmet needs declined from 2018 to 2020 across all subgroups.

Supplementary Table S2 shows the sociodemographic distribution of unmet medical needs among Korean nationals, which served as the reference population for SPR comparisons. Similar to the immigrant population, the prevalence of unmet medical needs was consistently higher among non-working individuals and women in 2018 and 2020. The overall prevalence decreased over time in most subgroups.

As shown in Table 1, working immigrants had lower SPRs than working Korean nationals that were below 1.0 in both the years: 0.879 (95% confidence interval [CI]: 0.872–0.886) in 2018 and 0.745 (95% CI: 0.738–0.751) in 2020. Conversely, non-working immigrants showed higher SPRs than their Korean counterparts that were above 1.0 in both the years: 1.117 (95% CI: 1.106–1.127) in 2018 and 1.128 (95% CI: 1.117–1.139) in 2020. This pattern remained consistent over time. Although the relative prevalence among working immigrants further decreased in 2020 (*p* for difference < 0.001), the excess risk among non-working individuals persisted with minimal change from 2018 (*p* for difference = 0.1199).

**Figure 2:**
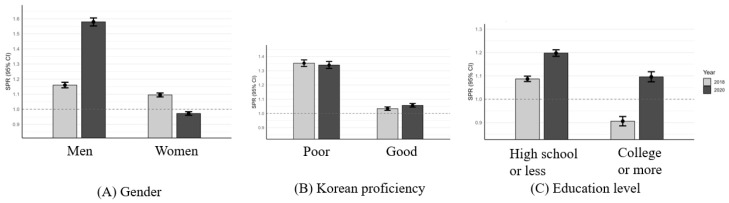
Standardized prevalence ratios (SPRs) of unmet medical needs among non-working immigrants in Korea by gender (**A**), Korean proficiency (**B**), and education level (**C**), 2018 and 2020. Note. SPRs were calculated by indirect standardization using the general Korean population from the Korea National Health and Nutrition Examination Survey (KNHANES) as reference. Error bars represent 95% confidence intervals. The horizontal dotted line at 1.0 indicates the reference value where the observed prevalence equals the expected prevalence. All ratios compare non-workers among immigrants with non-workers among Korean nationals. Stratified comparisons were made as follows: (**A**) Gender: Male and female SPRs were standardized separately against non-working Korean men and women, respectively. (**B**) Korean proficiency: All respondents were compared against the total non-working Korean population due to lack of stratified reference data. (**C**) Education level: Respondents with high school education or less were compared with non-working Koreans of the same educational level; those with college or more were compared with their similarly educated Korean counterparts. Abbreviations: SPR, standardized prevalence ratio; CI, confidence interval; KNHANES, Korea National Health and Nutrition Examination Survey.

Figure 2 presents the SPRs for unmet medical needs among non-working immigrants in Korea, stratified by gender, Korean language proficiency, and education level in 2018 and 2020. In 2018, male immigrants had an SPR of 1.160 (95% CI: 1.142–1.179), which was slightly higher than that of female immigrants (1.095; 95% CI: 1.082–1.108). By 2020, the SPR for men increased sharply to 1.579 (95% CI: 1.552–1.605), whereas the SPR for women decreased to 0.972 (95% CI: 0.960–0.984). Further, individuals with poorer Korean proficiency had consistently higher SPRs than those with good proficiency in both survey years. The SPRs were 1.353 (95% CI: 1.330–1.376) and 1.034 (95% CI: 1.022–1.046) and 1.340 (95% CI: 1.316–1.365) and 1.057 (95% CI: 1.045–1.070) in 2018 and 2020, respectively. In 2018, the SPRs differed by education level: those with a high school education or less showed a significantly elevated risk (1.087; 95% CI: 1.076–1.099), whereas those with a college education or higher showed a significantly lower risk (0.906; 95% CI: 0.886–0.926). In 2020, the SPRs increased in both groups. Among those with a high school education or lower, the risk was further elevated (1.198; 95% CI: 1.184–1.212). Among those with a college education or higher, the SPR increased above 1.0, indicating a shift to a statistically significant excess risk (1.096; 95% CI: 1.075–1.118).

All stratified comparisons (e.g., by gender, Korean proficiency, and education level) showed statistically significant differences between subgroups and across survey years (2018 vs. 2020), based on the Byar test (*p* < 0.05), except for the “Good Korean proficiency” group, where no significant difference was observed between 2018 and 2020 (*p* = 0.31).

**Figure 3:**
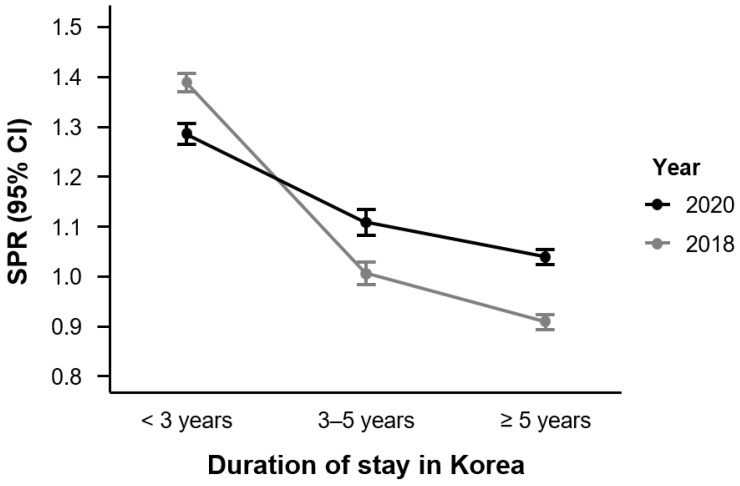
Standardized prevalence ratios (SPRs) of unmet medical needs among non-working immigrants in Korea, by duration of stay, 2018 and 2020. Note. SPRs were calculated by indirect standardization, comparing non-working immigrants to non-working Koreans from the Korea National Health and Nutrition Examination Survey (KNHANES), which served as the reference population. Duration of stay was categorized as under 3 years, 3–5 years, and more than 5 years. Error bars represent 95% confidence intervals. Abbreviations: SPR, standardized prevalence ratio; CI, confidence interval; KNHANES, Korea National Health and Nutrition Examination Survey.

Figure 3 illustrates the SPRs for unmet medical needs among non-working immigrants in Korea, stratified by duration of stay that was categorized as <3, 3–5, and ≥5 years. In 2018, the SPR for those who had stayed in Korea for <3 years was 1.390 (95% CI: 1.371–1.409), followed by 1.007 (95% CI: 0.985–1.029), 3–5 years, and 0.910 (95% CI: 0.895–0.925), ≥5 years. In 2020, the corresponding SPRs were 1.286 (95% CI: 1.265–1.308), 1.108 (95% CI: 1.083–1.135), and 1.039 (95% CI: 1.024–1.054), respectively. Compared with non-working Koreans, non-working immigrants showed a decreasing trend in SPRs with a longer duration of stay in both years. Notably, in 2020, the SPR exceeded 1.0 in all duration groups, whereas the longest-stay group had an SPR below 1.0 in 2018.

Supplementary Table S3 presents the weighted percentage distribution of the primary reasons for unmet medical needs among non-working immigrants in Korea by year and subgroup. In 2018 and 2020, cost burden was the most frequently reported reason overall (2018: 41.0%; 2020: 38.3%), followed by language barriers (2018: 26.4%; 2020: 25.9%) and lack of time (2018: 19.5%; 2020: 21.1%). The proportion reporting being “unaware of where to go” was relatively low in both years (2018: 5.2%; 2020: 3.4%). When stratified by gender, male respondents reported language barriers more frequently (2018: 33.0%; 2020: 31.6%) than female respondents (2018: 20.1%; 2020: 20.2%). In contrast, the cost burden was more frequently cited by women (2018: 46.6%; 2020: 41.7%). Those with poor Korean proficiency most frequently cited language barriers as the main reason (2018: 56.0%; 2020: 58.6%), whereas those with good proficiency reported cost burden more often (2018: 47.8%; 2020: 46.6%). Respondents with a high school education or less most frequently cited the cost burden (2018: 44.0%; 2020: 41.3%), whereas those with a college education or more frequently reported time constraints (2018: 23.8%; 2020: 23.5%). Further, language barriers were more frequently cited by those residing for <3 years (2018: 48.8%; 2020: 49.1%), whereas cost burden was the most common reason among those with 3–5 years (2018: 43.1%; 2020: 35.7%) or ≥5 years (2018: 52.3%; 2020: 45.9%) of residence.

Supplementary Table S4 presents the age-specific prevalence estimates of unmet medical needs according to nationality and year. The prevalence among foreign men decreased across all age groups between 2018 and 2020. In particular, among Korean men, the 30–39 age group showed a sharp decrease from 16.7% to 4.9% and the 50–59 age group from 8.4% to 3.1%. In contrast, among foreign men, the 30–39 age group showed a slight increase from 7.9% to 8.9%, while the 50–59 age group showed a decrease from 10.7% to 7.9%. In 2018, the prevalence of unmet medical needs among women was similar among foreign and Korean nationals across most age groups. In 2020, the prevalence among foreign women decreased or remained stable across all age groups, whereas the prevalence among Korean women did not show substantial changes. Consequently, the relative differences between the two groups narrowed, and in several age groups, the prevalence among foreign women was slightly lower than that among their Korean counterparts.

To assess the robustness of SPR estimates, we conducted a sensitivity analysis using the Poisson approximation method for confidence interval estimation (Supplementary Table S5). The results were largely consistent with those obtained using the Byar method across all analytical subgroups, supporting the stability of our findings.

## 4. Discussion

This study examined disparities in unmet medical needs among immigrants in Korea across the pre-pandemic (2018) and early-pandemic (2020) periods. We found that the SPRs remained lower among workers and higher among non-workers, with the gap widening over time. Subgroup analyses also indicated greater unmet needs among men and those with poor Korean proficiency, with lower education, and shorter durations of stay. The magnitudes and directions of these disparities changed heterogeneously between 2018 (pre-pandemic) and 2020 (early pandemic).

These findings can be interpreted through the Andersen Behavioral Model [8]. The persistent excess risk of unmet medical needs among non-working immigrants reflects a systematic deficit in enabling resources, particularly insurance coverage and income tied to employment status, rather than differences in predisposing characteristics or perceived need alone. Korean language proficiency and educational level, operating as predisposing characteristics, shaped individuals’ capacity to navigate the health system, while duration of stay served as a marker of acculturative integration, reflecting progressive shifts in both predisposing and enabling domains over time. The pandemic functioned as an external shock that further constrained enabling resources across the immigrant population, while disproportionately exposing those whose enabling resources were already structurally limited, most notably non-working men, recent arrivals, and those with limited Korean proficiency.

Workers had lower relative risks than Koreans, reflecting the healthy migrant effect and the role of employment-linked health insurance and screening systems [32,33,34,35]. In contrast, non-working immigrants had elevated unmet needs, likely due to lower insurance coverage, lower socioeconomic status, and reduced institutional integration [6,10,36]. During the early pandemic, this divide widened, as workers may have actively avoided healthcare, a pattern consistent with prior evidence that pandemic-related fear of quarantine discouraged health care-seeking among those in precarious employment [37], while non-working immigrants were more affected by reduced service availability and ongoing structural barriers.

In the subgroup analyses, male immigrants consistently exhibited higher SPRs than women in both periods. Men more frequently reported language and cost barriers, whereas women primarily cited financial constraints. Additionally, women report lower rates of being unaware of where to seek care, which may reflect the stronger familial support networks commonly available to married migrant women, facilitating better navigation of the healthcare system [38]. The increase in SPRs among foreign men in 2020 appears largely relative, as healthcare utilization declined sharply among Korean men during the pandemic due to infection concerns and service disruptions [15,37]. In contrast, the decrease in SPRs among foreign women suggests that their unmet needs declined at a rate comparable to or exceeding that of Korean women. This pattern may be attributed to stronger familial and social support networks among female marriage migrants, which may have buffered the impact of pandemic-related disruptions [3]. Thus, the widening gender gap reflects differential vulnerabilities to system-level shocks rather than abrupt deterioration in immigrant men’s access.

Korean language proficiency remained a persistent determinant of unmet medical needs [1,2]. Those with poor Korean proficiency consistently exhibited higher SPRs than those with good proficiency in both 2018 and 2020. Importantly, this disparity remained stable during the early pandemic period, suggesting that language barriers served as a persistent structural obstacle to healthcare access irrespective of broader pandemic-related disruptions.

Consistent with the general expectation that education reflects the SES, individuals with lower educational levels exhibited higher SPRs for unmet medical needs in our study, and financial burden emerged as a major barrier among these groups. Notably, during the early pandemic period, even among immigrants with relatively higher education levels, who might have been expected to have greater resources and resilience, the SPR for unmet medical needs increased. The pandemic may have disrupted traditional protective factors and exposed vulnerabilities even among those showing higher-SES indicators. Similar patterns have been noted in broader pandemic research [16]. Thus, although lower-SES groups remained at a heightened risk, higher-educated immigrants were no longer fully shielded from barriers to healthcare access during the pandemic. This financial vulnerability may also be shaped by the institutional context of the 2019 reform of the National Health Insurance system, which introduced mandatory enrollment alongside standardized, average-based premium requirements. Although designed to expand formal coverage, such requirements may have imposed additional burdens on economically inactive immigrants, particularly since even brief arrears can result in the immediate restriction of benefits and potential disadvantages in visa renewals. This institutional framework could have reinforced cost-related barriers during the early pandemic, potentially encouraging voluntary avoidance of healthcare utilization to avoid further financial or legal risks.

Duration of stay was another key determinant. SPRs were highest among recent immigrants and declined with longer residence, indicating improved adaptation and integration over time. Notably, recent immigrants cited language barriers most frequently, whereas long-term residents reported financial issues as dominant obstacles, suggesting that while language and system unfamiliarity dominate in the early phase of settlement [1,7], these barriers tend to diminish over time, with financial and structural constraints emerging as the primary obstacles in later stages [39]. However, in 2020, even long-term residents experienced elevated SPRs, indicating that extended stay does not fully protect against system-wide shocks, consistent with prior observations [7,16].

These findings have several policy implications. The persistent disparities among non-working immigrants suggest the need to review current eligibility structures for health insurance coverage, particularly for those outside formal employment arrangements. The stability of language-related barriers across both study periods underscores the importance of expanding language support services within the healthcare system. Furthermore, the widening of disparities during the early pandemic period indicates that existing safety nets may be insufficient during public health emergencies, highlighting the need for more inclusive contingency measures that extend beyond employment-based frameworks.

To our knowledge, this is the first study using nationally representative data to compare unmet medical needs between immigrants and non-immigrants during pre- and early-pandemic periods. The use of official national datasets and robust methods strengthens the generalizability of our findings. Limitations include reliance on self-reported unmet needs. While this is a widely accepted pragmatic indicator in global population surveys, it cannot capture the full multidimensionality of healthcare barriers and may introduce measurement biases such as recall error. Importantly, cultural differences in reporting thresholds for unmet medical needs may affect comparability across immigrant subgroups, immigrants from cultures with higher tolerance for untreated illness or greater reluctance to self-identify unmet needs may systematically underreport barriers, introducing immigrant perception bias [40]. These considerations suggest that our estimates may represent a conservative lower bound of true unmet need disparities. Also, the outcome measures reflects access-based unmet needs and does not capture quality-of-care dimensions, such as hospital readmission rates, which represent adequacy of care received once access has been obtained. Nevertheless, the consistent and statistically significant disparities identified across all subgroups confirm that this access-focused measure is both valid and sensitive for detecting structural healthcare inequalities among immigrant populations. Furthermore, while the overall sample size was large, stratification in our subgroup analyses inherently reduced the number of observations within specific demographic categories. This reduction may have widened the confidence intervals and limited the statistical power to detect smaller meaningful differences within certain subgroups. Additionally, because these national surveys are based on official registries, undocumented groups were not included in the analysis, which may affect the representativeness of findings for the most marginalized segment of the immigrant population. Although standardization was adjusted for age and gender, other potential confounders, such as health status and healthcare-seeking behaviors, were not fully controlled, potentially resulting in residual confounding. Although Korea’s structured immigration system offers unique insights, these findings may not be generalizable to countries with different immigration and healthcare systems.

In conclusion, this study highlights the persistent and widening disparities in unmet medical needs among non-working immigrants in Korea, particularly among men, those with poor Korean language proficiency, and those with shorter durations of stay.

## 5. Conclusions

This study shows that inequalities in unmet medical needs between immigrants and native-born populations in Korea persisted from the pre-pandemic to early-pandemic period, with employment status functioning as a key structural determinant of access to care. Non-working immigrants consistently experienced elevated unmet needs, particularly among men, those with limited Korean proficiency, lower education, and shorter durations of stay. The findings indicate that unmet medical needs among immigrants reflect structural constraints embedded in labor-oriented immigration and healthcare systems. Strengthening healthcare equity for immigrant populations requires policy approaches that go beyond employment-based coverage and address language access, insurance inclusion, and system navigation, especially during periods of societal disruption.

## Figures and Tables

**Table 1:** Standardized prevalence ratios for unmet medical needs by work status among immigrants in Korea, 2018 and 2020.

Work Status	2018	2020
Workers	0.879 (0.872–0.886)	0.745 (0.738–0.751)
Non-workers	1.117 (1.106–1.127)	1.128 (1.117–1.139)

Note: SPRs were calculated using indirect standardization based on age and gender; 95% confidence intervals (CIs) were estimated using Byar’s method. Korean nationals from the Korea National Health and Nutrition Examination Survey (KNHANES) were used as the reference population. Immigrant workers were compared to Korean workers, and non-workers were compared to their Korean counterparts. SPR, standardized prevalence ratio; CI, confidence interval.

## Data Availability

KNHANES data are publicly accessible (https://knhanes.kdca.go.kr; accessed on 2 April 2026). Data from the Survey on Immigrants’ Living Conditions and Labor Force are available upon request through Statistics Korea’s MDIS system (https://mdis.kostat.go.kr; accessed on 2 April 2026).

## Supplementary Materials

The following supporting information can be downloaded at: https://www.mdpi.com/article/10.3390/healthcare14091226/s1, Table S1: Characteristics by Experience of Unmet Medical Needs among Foreign Nationals in Korea, 2018 and 2020; Table S2: Sociodemographic Characteristics by Experience of Unmet Medical Needs among the General Population in Korea, 2018 and 2020; Table S3: Weighted Percentage Distribution of Reasons for Unmet Medical Needs among Unemployed Foreign Nationals, by Year and Subgroup; Table S4: Weighted Prevalence (%) of Unmet Medical Needs by Age Group, Gender, and Nationality in 2018 and 2020; Table S5: Sensitivity Analysis Using Poisson-Based Standardized Prevalence Ratios.
