# Supplementary material for: Unmet Medical Needs Among Immigrants in Korea Before and During COVID-19
Source: Healthcare (Basel). 2026 May 2;14(9):1226. doi: 10.3390/healthcare14091226 (PMC13163738; doi:10.3390/healthcare14091226)
Supplement: Supplementary file 1 [file healthcare-14-01226-s001.zip › healthcare-4256543-supplementary.pdf]

Supplementary Materials

Supplementary Table S1. Characteristics by Experience of Unmet Medical Needs among Foreign Nationals in Korea, 2018 and 2020.

Supplementary Table S2. Sociodemographic Characteristics by Experience of Unmet Medical Needs among the General Population in Korea, 2018 and 2020

Supplementary Table S3. Weighted Percentage Distribution of Reasons for Unmet Medical Needs among Unemployed Foreign Nationals, by Year and Subgroup

Supplementary Table S4. Weighted Prevalence (%) of Unmet Medical Needs by Age Group, Gender, and Nationality in 2018 and 2020

Supplementary Table S5. Sensitivity Analysis Using Poisson-Based Standardized Prevalence Ratios.

Supplementary Table S1. Characteristics by Experience of Unmet Medical Needs among Foreign Nationals in Korea, 2018 and 2020.

Characteristics	2018			2020		
	No unmet medical needs	Unmet medical needs	p-value	No unmet medical needs	Unmet medical needs	p-value
Work status						
Workers	810362 ± 3081 (93.1 ± 0.4%)	59672 ± 3081 (6.9 ± 0.4%)	<0.0001	773416 ± 2322 (94.2 ± 0.3%)	47405 ± 2322 (5.8 ± 0.3%)	0.0003
Non-workers	388405 ± 2415 (90.2 ± 0.6%)	42312 ± 2415 (9.8 ± 0.6%)		472687 ± 2013 (92.5 ± 0.4%)	38306 ± 2013 (7.5 ± 0.4%)	
Age group						
15-29	358416 ± 2092 (92.5 ± 0.5%)	29258 ± 2092 (7.5 ± 0.5%)	0.0027	358101 ± 1763 (93.1 ± 0.5%)	26706 ± 1763 (6.9 ± 0.5%)	0.0002
30-39	327509 ± 1835 (93.6 ± 0.5%)	22436 ± 1835 (6.4 ± 0.5%)		354592 ± 1497 (94.8 ± 0.4%)	19392 ± 1497 (5.2 ± 0.4%)	
40-49	194288 ± 1735 (91.4 ± 0.8%)	18292 ± 1735 (8.6 ± 0.8%)		198358 ± 1156 (94.5 ± 0.6%)	11529 ± 1156 (5.5 ± 0.6%)	
50-59	195780 ± 1653 (91.8 ± 0.8%)	17505 ± 1653 (8.2 ± 0.8%)		191665 ± 1268 (92.6 ± 0.6%)	15212 ± 1268 (7.4 ± 0.6%)	
≥60	122775 ± 1358 (89.4 ± 1.0%)	14493 ± 1358 (10.6 ± 1.0%)		143387 ± 1071 (91.8 ± 0.7%)	12872 ± 1071 (8.2 ± 0.7%)	
Gender						
Men	691443 ± 2833 (93.1 ± 0.4%)	51154 ± 2833 (6.9 ± 0.4%)	0.0003	707140 ± 2253 (94.2 ± 0.3%)	43587 ± 2253 (5.8 ± 0.3%)	0.0019
Women	507325 ± 2704 (90.9 ± 0.5%)	50831 ± 2704 (9.1 ± 0.5%)		538963 ± 2091 (92.8 ± 0.4%)	42124 ± 2091 (7.2 ± 0.4%)	
Korean proficiency						
Poor	270706 ± 2014 (90.5 ± 0.7%)	28255 ± 2014 (9.5 ± 0.7%)	0.0032	299492 ± 1786 (91.9 ± 0.5%)	26439 ± 1786 (8.1 ± 0.5%)	0.0001
Good	928062 ± 3361 (92.6 ± 0.3%)	73729 ± 3361 (7.4 ± 0.3%)		946610 ± 2496 (94.1 ± 0.2%)	59272 ± 2496 (5.9 ± 0.2%)	
Education level						

≤High school	870782 ± 3441 (91.7 ± 0.4%)	79283 ± 3441 (8.3 ± 0.4%)	0.0061	873820 ± 2664 (93.2 ± 0.3%)	63578 ± 2664 (6.8 ± 0.3%)	0.0187
≥college	327986 ± 1875 (93.5 ± 0.5%)	22702 ± 1875 (6.5 ± 0.5%)		372282 ± 1532 (94.4 ± 0.4%)	22134 ± 1532 (5.6 ± 0.4%)	
Duration of stay in Korea						
<3 years	257661 ± 1848 (91.9 ± 0.7%)	22634 ± 1848 (8.1 ± 0.7%)	0.1145	229442 ± 1250 (93.7 ± 0.5%)	15441 ± 1250 (6.3 ± 0.5%)	0.0216
3-5 years	401119 ± 2316 (91.4 ± 0.5%)	37623 ± 2316 (8.6 ± 0.5%)		358331 ± 1893 (92.6 ± 0.5%)	28680 ± 1893 (7.4 ± 0.5%)	
≥5 years	539988 ± 2566 (92.8 ± 0.4%)	41727 ± 2566 (7.2 ± 0.4%)		658330 ± 2070 (94.1 ± 0.3%)	41591 ± 2070 (5.9 ± 0.3%)	

Notes. Weighted frequencies and percentages are presented. P-values were obtained from chi-squared tests using survey weights.

Supplementary Table S2. Sociodemographic Characteristics by Experience of Unmet Medical Needs among the General Population in Korea, 2018 and 2020

Characteristics	2018			2020		
	No unmet medical needs	Unmet medical needs	p-value	No unmet medical needs	Unmet medical needs	p-value
Work status						
Workers	25020729±807192 (91.8±0.5)	2222942±157180 (8.2±0.5)	0.5993	23910597±917833 (93.2±0.5)	1736369±134583 (6.8±0.5)	0.7877
Non-workers	14442241±514840 (91.4±0.7)	1355692±115606 (8.6±0.7)		15316088±621554 (93.4±0.6)	1074454±104022 (6.6±0.6)	
Age group						
15-29	8815126±508270 (91.8±1.0)	792555±105788 (8.2±1.0)	0.8369	8683256±523608 (94.3±0.8)	529165±81078 (5.7±0.8)	0.6662
30-39	6610660±373023 (91.9±1.1)	582462±91069 (8.1±1.1)		6302782±393399 (92.3±1.4)	528910±107139 (7.7±1.4)	
40-49	7485454±418768 (92.5±0.9)	611217±77657 (7.5±0.9)		7250317±442637 (93.1±0.9)	534422±76162 (6.9±0.9)	
50-59	7313545±348430 (91.7±1.0)	662262±84887 (8.3±1.0)		7578608±425165 (93.7±1.0)	509619±83698 (6.3±1.0)	
≥60	9238185±424989 (90.9±0.9)	930138±97713 (9.1±0.9)		9411722±455109 (93.0±0.6)	708707±67333 (7±0.6)	
Gender						
Men	19974439±709393 (93.5±0.5)	1391489±120173 (6.5±0.5)	<.0001	20083754±725333 (94.9±0.5)	1079423±106905 (5.1±0.5)	0.0001
Women	19488530±589541 (89.9±0.6)	2187144±144932 (10.1±0.6)		19142932±673596 (91.7±0.7)	1731400±137979 (8.3±0.7)	
Education level						
≤High school	15039731±694152 (92.5±0.7)	1228088±126575 (7.5±0.7)	0.1731	15905578±849826 (93.6±0.7)	1087410±126434 (6.4±0.7)	0.5783
≥college	24417554±791083 (91.2±0.6)	2350546±170969 (8.8±0.6)		23321107±853546 (93.1±0.5)	1723414±139210 (6.9±0.5)	

Notes: Weighted frequencies and percentages are reported with standard errors (± SE). P-values were obtained from chi-squared tests using

survey weights in the Korea National Health and Nutrition Examination Survey (KNHANES).

Supplementary Table S3. Weighted Percentage Distribution of Reasons for Unmet Medical Needs among Unemployed Foreign Nationals, by Year and Subgroup

	2018					2020				
	Language barrier	Cost burden	Unaware of where to go	No time	Other	Language barrier	Cost burden	Unaware of where to go	No time	Other
Overall	26.4	41.0	5.2	19.5	7.9	25.9	38.3	3.4	21.1	11.4
Gender										
Men	33	35.1	6.5	17.8	7.5	31.6	34.8	4.3	19.0	10.3
Women	20.1	46.6	3.9	21.1	8.3	20.2	41.7	2.5	23.1	12.6
Korean proficiency										
Good	15.4	47.8	4.7	23	9.1	11.8	46.6	3.6	25.3	12.8
Poor	56	22.7	6.5	9.9	4.8	58.6	18.9	2.9	11.3	8.4
Education level										
≤High school	25.1	44.0	5.0	18.2	7.8	24.9	41.3	3.7	20.2	9.9
≥College	31.3	30.4	6.0	23.8	8.5	28.8	29.3	2.5	23.5	15.9
Duration of stay in Korea										
<3 years	48.8	26.3	6.2	12.7	6.0	49.1	27.9	3.5	12.0	7.5
3-5 years	22.0	43.1	6.0	18.4	10.5	27.5	35.7	4.0	15.5	17.2
≥5 years	9.7	52.3	3.9	25.8	8.3	10.2	45.9	3.1	28.9	11.9

Notes. Percentages were weighted and calculated among non-working foreign nationals who reported experiencing unmet medical needs in the past 12 months. Respondents were asked to indicate the primary reason for being unable to seek medical care.

Supplementary Table S4. Weighted Prevalence (%) of Unmet Medical Needs by Age Group, Gender, and Nationality in 2018 and 2020

Year	Population	Gender	15–29	30–39	40–49	50–59	≥60
2018	Foreign	Men	8 ± 1.3%	7.9 ± 2.3%	15.6 ± 4.1%	10.7 ± 2.4%	8.9 ± 2%
		Women	9.2 ± 1.1%	8.8 ± 1.5%	10 ± 2.1%	10.8 ± 2%	12.8 ± 1.8%
2018	Korean	Men	6.2±1.6	16.7±7.1	5.7±4.2	8.4±5.8	5.6±1.2
		Women	7.2±1.8	10.6±2.7	9.2±2.4	10.1±2.4	10.8±1.4
2020	Foreign	Men	6.1 ± 1%	8.9 ± 1.8%	7.5 ± 2.4%	7.9 ± 1.6%	7.9 ± 1.4%
		Women	6.8 ± 0.8%	5.5 ± 0.9%	7.4 ± 1.4%	8.5 ± 1.5%	11.2 ± 1.3%
2020	Korean	Men	4.2±1.2	4.9±4.7	9.3±4.9	3.1±2.5	3.3±0.8
		Women	6.5±2.1	10.4±3.7	6.0±1.7	5.7±1.7	10.3±1.3

Notes. Values are presented as weighted prevalence ± standard error (SE), expressed as percentages. Data were stratified by gender and age group (15–29, 30–39, 40–49, 50–59, and ≥60 years). Estimates are based on the Korea National Health and Nutrition Examination Survey (KNHANES) for Korean nationals and the Survey on Immigrants' Living Conditions and Labour Force for foreign nationals.

Supplementary Table S5. Sensitivity Analysis Using Poisson-Based Standardized Prevalence Ratios.

Population	2018 SPR (95% CI)	2020 SPR (95% CI)
Workers	0.879 (0.872–0.886)	0.745 (0.738–0.751)
Non-workers	1.117 (1.106–1.127)	1.128 (1.117–1.139)
Non-workers & Men	1.16 (1.142–1.179)	1.579 (1.552–1.605)
Non-workers & Women	1.095 (1.082–1.108)	0.972 (0.96–0.984)
Non-workers & Poor Korean proficiency	1.353 (1.33–1.376)	1.34 (1.316–1.365)
Non-workers & Good Korean proficiency	1.034 (1.022–1.046)	1.057 (1.045–1.07)
Non-workers & High school or less	1.087 (1.076–1.099)	1.198 (1.184–1.212)
Non-workers & College or more	0.906 (0.886–0.926)	1.096 (1.075–1.118)
Non-workers & <3 yrs	1.39 (1.371–1.409)	1.286 (1.265–1.308)
Non-workers & 3–5 yrs	1.007 (0.985–1.029)	1.108 (1.083–1.135)
Non-workers & ≥5 yrs	0.91 (0.895–0.925)	1.039 (1.024–1.054)

Notes: SPRs and 95% confidence intervals were calculated using the Poisson approximation method. .All stratified comparisons (e.g., by gender, Korean proficiency, and education level) showed statistically significant differences between subgroups and across survey years (2018 vs 2020), based on the Byar test ($p < 0.05$), except for the "Good Korean proficiency" group, where no significant difference was observed between 2018 and 2020 ($p = 0.45$)
Abbreviations: SPR, standardized prevalence ratio; CI, confidence interval
